# Health economic evaluation of a nurse-assisted online eye screening in home healthcare to reduce avoidable vision impairment (iScreen): study protocol for a cluster randomized controlled trial

**DOI:** 10.1186/s13063-023-07882-0

**Published:** 2024-02-02

**Authors:** Vera Rooth, Hilde van der Aa, Robert P. L. Wisse, Otto R. Maarsingh, Marc Koopmanschap, Jan E. E. Keunen, Hester Vermeulen, Caroline C. W. Klaver, Gabriëlle Janssen, Ger H. M. B. van Rens, Ruth M. A. van Nispen

**Affiliations:** 1 Department of Ophthalmology, Vrije Universiteit Amsterdam, Amsterdam UMC, Amsterdam, The Netherlands; 2 Quality of Care, Aging and Later Life, Health Behaviors and Chronic Diseases, Amsterdam Public Health, Amsterdam, The Netherlands; 3 Department of Ophthalmology, UMC Utrecht, Utrecht, The Netherlands; 4 General Practice, Amsterdam Public Health, Vrije Universiteit Amsterdam, Amsterdam UMC, Amsterdam, The Netherlands; 5 Erasmus School of Health Policy & Management, Health Technology Assessment (HTA), Erasmus University Rotterdam, Rotterdam, The Netherlands; 6 Department of Ophthalmology, Radboudumc, Nijmegen, The Netherlands; 7 Radboud Institute for Health Sciences, IQ Healthcare, Radboudumc, Nijmegen, The Netherlands; 8 Department of Ophthalmology, Erasmus MC, Rotterdam, The Netherlands; 9 Institute of Molecular and Clinical Ophthalmology Basel, Basel, Switzerland; 10 Optometristen Vereniging Nederland, Utrecht, The Netherlands

**Keywords:** Eye screening, Older adults, Home healthcare, Vision impairment, Cluster randomized controlled trial

## Abstract

**Background:**

Among older people undiagnosed and untreated vision impairment and blindness are common. The leading causes are uncorrected refractive errors and cataracts. Vision problems are associated with a lower quality of life, several health problems, and a higher chance of falling accidents and fractures. To eliminate avoidable vision impairment and blindness, targeted eye screening programs are recommended. Older patients, receiving home healthcare, have not yet been considered as a population at risk who could benefit from eye screening.

**Methods:**

A cluster-randomized controlled trial will be conducted to investigate the cost-effectiveness and cost-utility of online nurse-assisted eye screening in home healthcare, compared to care as usual, in reducing avoidable vision impairment. A healthcare and societal perspective will be used. The study will be performed in collaboration with several home healthcare organizations in the Netherlands. The online eye screening consists of near and distance visual acuity, followed by an Amsler grading test. Measurements in both groups will take place at baseline and after 6 and 12 months of follow-up. A total of 240 participants will be recruited. Older men and women (65 +), who receive home-based nursing and are cognitively able to participate, will be included. The primary outcome will be the change of two lines or more on the Colenbrander-1 M visual acuity chart between baseline and 12-month follow-up.

**Discussion:**

An eye screening for populations at risk contributes to the detection of undiagnosed and untreated vision impairment. This may reduce the health-related consequences of vision loss and the high economic burden associated with vision impairment.

**Trial registration:**

ClinicalTrials.gov NCT06058637. Registered on 27 September 2023.

**Supplementary Information:**

The online version contains supplementary material available at 10.1186/s13063-023-07882-0.

## Background

Age is an important risk factor for vision impairment and blindness [1, 2]. Due to aging of the world population, an increase in older people with vision impairment is expected in the upcoming years [2–6]. In 2020, 1.1 billion people had distance vision impairment or uncorrected presbyopia worldwide. By 2050, this is expected to rise to 1.8 billion people [2]. The older population residing in care institutions and receiving home healthcare in the Netherlands experience a high prevalence of blindness and low vision, ranging from 20 to 25% [7, 8]. This is in line with the global prevalence of moderate to severe vision loss or blindness, for individuals aged 70 years or older, which is 22% and 5%, respectively [3], as well as self-reported data from nursing home facilities across eight European countries, reporting a 19.5% prevalence [9].

Vision impairment in older people is associated with lower quality of life and barriers to participate in society [5, 10–12]. It can also lead to health problems such as depressive symptoms, falls, and fractures [6, 12–17]. Furthermore, older adults with severe vision impairment can experience difficulties in obtaining health information due to the way the information is presented. Access to health information is necessary for making healthcare decisions and to follow up on healthcare recommendations [18].

Worldwide, the main causes of vision impairment and blindness are uncorrected refractive errors and cataracts. Other important causes are age-related macular degeneration, diabetic retinopathy, and glaucoma [19–21]. Although both leading causes of vision impairment and blindness are treatable with cost-effective interventions [3, 5, 21], uncorrected refractive errors and cataracts still make up approximately 41% and 39% of all moderate to severe vision impairment, respectively [20]. In a study by Limburg et al. cataract was even the main cause (51%) of vision impairment among residents in care institutions in the Netherlands [22]. It was found that up to 34% of older people with vision impairment could benefit from having appropriate spectacles [12]. Another study demonstrated improvement in quality of life and depressive symptoms in adults living in nursing homes who received spectacle prescriptions within 2 months [23]. Cataracts can be easily treated by surgically replacing the cloudy lens content with an artificial intraocular lens. Studies have shown that cataract surgery yields a positive effect on vision-related quality of life in older adults living in nursing homes [24], and it reduces the risk of falls among older adults [17, 25].

Despite the fact that the main causes of vision impairment have been well-known for many years, a high level of undiagnosed and untreated vision impairment among older people still exists [11]. To tackle this issue, the World Health Organization (WHO) recommends not only low- and middle-income countries, but also high-income countries to create awareness and establish eye-screening programs for the prevention of avoidable vision impairment and blindness [26]. This may reduce the high economic burden associated with vision impairment as well. It has been considered meaningful, given the direct and indirect care needs of visually impaired older adults, to detect any avoidable vision impairment in people in care institutions at an early stage [1, 8]. In addition, older adults will benefit longer when vision problems are detected at an early stage [1].

Patients receiving home healthcare, as a partly dependent population living in the community, have not been considered as a population at risk that could benefit from eye screening [7]. As of 2021, 8% of older adults in the Netherlands between the ages of 65 and 75 received home healthcare and 30% of people 75 years and older [27]. A systematic review by Clarke et al. [11] demonstrated that eye screening in a general practice setting did not improve vision among older adults due to the fact that they often did not follow up on the offered intervention as a result of the tests. They suggested further research to improve intervention uptake after screening, with a particular focus on healthcare-dependent populations rather than focusing solely on low-risk community groups [11]. In turn, a recent cross-sectional pilot study in one of the largest home healthcare organizations in the Netherlands showed that simple eye screening by community nurses can help detect eye problems among a potentially vulnerable older population still living in a relatively independent setting [7]. Approximately 20% of the patients were referred for eye problems that had not been diagnosed previously, and of those, only half of them actually used their referral to go to their general practitioner (GP), an optician, optometrist, or ophthalmologist. Half of them presented with severe vision impairment. In most cases, the eye problems could be corrected with spectacles or cataract surgery. The study highlighted that without eye screening in the home healthcare setting, many eye problems would have been left undetected and untreated [1].

Considering the common issues with mobility among this older population, receiving home healthcare, and hence difficulties to visit care facilities, utilizing e-health tools for eye screening in the home setting can be particularly beneficial. Previous research demonstrated good agreement between a nurse-assisted online eye screening and traditional visual acuity measurements, in a home healthcare population [28].

As the prevalence of visual problems seemed high and the impact of visual problems in the older population receiving home healthcare indicated unfavorable additional health outcomes, eye screening can be an important preventive measure in this vulnerable population. Moreover, as diagnostic and treatment options are available, screening tools are reliable, the natural course of eye diseases can often be predicted, the need for screening has been acknowledged, and the population at risk has been determined; it seems evident, considering the WHO relevance criteria for screening for potential health issues [29] that eye screening should be considered. However, an important aspect to assure the relevance of eye screening and its subsequent intervention uptake according to these criteria has not yet been investigated. There are no studies available regarding cost-utility and cost-effectiveness explaining the benefits of screening from a societal perspective in terms of relevant health indicators. As part of a larger study in which we also study the individual, healthcare, and socio-political context of eye screening in home healthcare, we present a protocol of a cluster-randomized controlled trial (RCT) to investigate the cost-effectiveness and cost-utility of “iScreen,” a nurse-assisted online eye screening (in addition to usual care) in home healthcare settings, compared to care as usual (CAU), in reducing avoidable vision impairment.

## Methods

### Study design and ethical approval

We will perform a cluster RCT to compare online eye screening, in addition to CAU, versus CAU. The eye screening will be guided by community nurses. Cost-effectiveness and cost-utility will be studied from a healthcare and societal perspective, including the impact on physical and mental health. We will map healthcare costs over a period of 1 year. The study will be performed in collaboration with three Dutch home healthcare organizations. The study protocol was approved by the Medical Ethics Committee of Amsterdam University Medical Centers (Amsterdam UMC), location VUmc in Amsterdam, the Netherlands. This study protocol was written according to the “Standard Protocol Items: Recommendations for Interventional Trial” (SPIRIT) reporting guidelines [30]. The SPIRIT checklist is added as an Additional file 1.

### Patient involvement

Stakeholder perspectives have been explored to investigate willingness to incorporate eye screening. In our recent qualitative exploratory study, interviews with professionals (*n* = 22) and patients (*n* = 8) were conducted to gain insight into barriers, facilitators, and public support for the implementation of an online eye screening (Aa van der H, Nassau van F, Elsman EBM, Wisse RPL, Maarsingh OR, Keunen J, et al: Facilitators and barriers for implementation of online nurse-assisted eye screening in home healthcare: a qualitative study, unpublished). Professionals and patients were optimistic about implementing the online eye screening and expressed its added value. They also provided relevant information on how to offer eye screening within home healthcare (e.g., by offering tailored guidance, improving user-friendliness, and providing clear referral trajectories), which we used to optimize our intervention (Aa van der H, Nassau van F, Elsman EBM, Wisse RPL, Maarsingh OR, Keunen J, et al: Facilitators and barriers for implementation of online nurse-assisted eye screening in home healthcare: a qualitative study, unpublished).

At the 6 month time period of the RCT, all patients from the intervention group (*n* ≈ 120) will be involved in an extensive process evaluation using elements of the RE-AIM framework [31] and the Telemonitoring Acceptance Framework [32]. Patients will get the opportunity to share their experience with the online eye screening and the referral process.

If the eye screening proves to be cost-effective, the results of this study and previous research will be presented during an invitational conference with important stakeholders, of whom at least five patient representatives. We will actively discuss how to overcome the known barriers and to use facilitators. Together with stakeholders, we will formulate the final implementation strategy and operational plan.

### Sample size

**Fig. 1:**
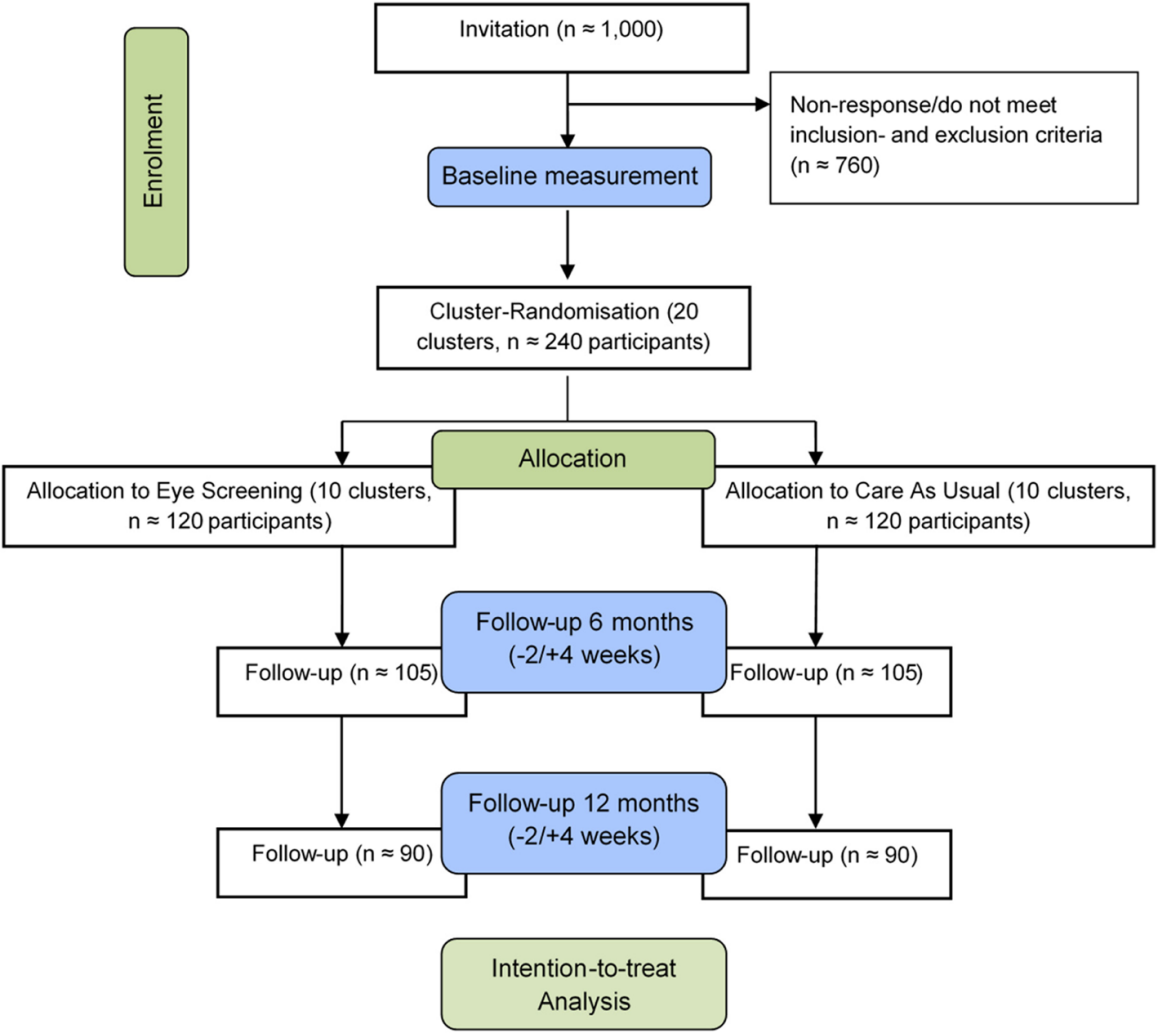
Flowchart of the iScreen study

Taking into account a potential dropout of one-third, the sample size calculation revealed that we need 120 participants in the intervention group (receiving eye screening in addition to CAU) and 120 participants in the control group (receiving only CAU). A team of community nurses who provide care to patients in a specific geographical area will form a cluster. We aim to include 10 clusters in the intervention group and 10 clusters in the control group, both with 12 subjects each (Fig. 1), but this may vary in practice depending on the willingness of patients to participate within one cluster. Based on these numbers, we will achieve 82% power to detect a difference between the group proportions of 0.15, having a clinically relevant change of two lines or more in visual acuity (primary outcome measure). The proportion in the intervention group is assumed to be 0.05 under the null hypothesis and 0.20 under the alternative hypothesis. The proportion in the control group is 0.05. The test statistic used was the two-sided *Z*-test (unpooled). The intracluster correlation was set at 0.02 [33], and the significance level of the test was set at 0.05. Based on previous research in older populations [34], we expect that we need to invite approximately 1000 patients to include 240 participants. Loss to follow-up of one-third means that 80 participants (approximately 40 in both trial arms) are allowed to dropout during the course of the study in order to estimate the abovementioned effect, which seems reasonable based on previous studies [34] (Fig. 1).

### Recruitment

Older patients from several home healthcare organizations in the Netherlands will be invited by their community nurse to participate. The community nurse will evaluate patients' eligibility using the inclusion and exclusion criteria (Table 1). Patients with terminal illnesses or patients receiving palliative home care will be excluded. Recruitment will take place in three waves by using three inclusion cycles between March 2023 and August 2024 or until the total number of study participants has been achieved. Potential participants may indicate their interest to participate in the trial by sending out a form with contact information to the research team at Amsterdam UMC. Next, the research team will contact them by telephone to answer questions about the study and inform the patient about the informed consent procedure. Patients who are interested in participating in the trial will sign the informed consent form. After providing informed consent, the additional inclusion and exclusion criteria (Table 1) will be assessed by the researcher by telephone and the baseline measurement will be performed.

**Table 1:** Inclusion and exclusion criteria

Inclusion criteria
- Patients receive home healthcare for health problems
- 65 years or older^a^
- Understanding of the Dutch language (telephone assessment)
- Cognitive ability to participate in research (telephone assessment: six-item Mini-Mental State Examination score > 3) [35]
Exclusion criteria
- Terminal illness, palliative home care^a^
- Cognitively unable to participate in research (e.g., late-stage Alzheimer’s, Parkinson’s (telephone assessment: six-item Mini-Mental State Examination score ≤ 3) [35]
- Having received an optometric or ophthalmic consultation within the last 6 months^a^ (telephone assessment)

^a^Criteria will be verified by a community nurse

### Randomization

Teams of community nurses (clusters), which will participate in the cluster RCT, will be randomized to either the intervention (eye screening in addition to CAU) or control group (CAU). As there are differences in the number of participants a team is able to include, we will group participating teams into matched pairs on the basis of number of participants to prevent imbalance between the group sizes. One researcher (VR) will use Castor to randomize the clusters, by using block randomization. Randomization of the clusters will be stratified by home healthcare organization to stimulate regional heterogeneity and to equally divide the additional burden on the organizations. This will be done after the community nurse has invited patients to participate in the study. Due to the nature of the intervention, community nurses and participants cannot be masked. Research assistants, who will perform visual acuity measurements and administer questionnaires during the follow-up assessments, will be masked. Participants are asked not to disclose the nature of their treatment allocation during the follow-up assessments. The research team will regularly check whether a research assistant is still masked by asking to guess to which trial arm a participant is allocated. Allocation to the intervention or control group will be communicated with the community nurses by email by an unmasked researcher.

### Intervention

On top of CAU, nurse-assisted eye screening will take place with the Easee test. This e-health tool was introduced to measure visual acuity without any physical eye chart, but instead using a computer, laptop or tablet, and a smartphone. The Easee test was originally developed for measuring visual acuity and spherical and cylindrical refractive errors in adults up to 45 years old. The test is Conformité Européenne marked and is also commercially available via the website of Easee (https://www.easee.online/nl/). A version of the Easee web tool has been tailored to the requirements of the older adults in this cluster RCT. Currently, the test is certified as a class 1 device under the Medical Device Directive 93/42/ECC. Class 2a certification is pending. Easee has an ISO13845 which is monitored and audited by TüV Rheinland, Germany. The software is classified as class A, in accordance with IEC 62304:2006.

The tests will be displayed on the laptop. The smartphone will function as a remote control by which the participant submits input to the laptop screen. If the participant can execute the test by him- or herself, the nurse will merely supervise to ensure correct application. If more assistance is needed, the nurse will provide this. Standard audio instructions guide the nurse and participant through the test. The test will start with four triage questions, which are used to set up a correct referral: (1) Are you diagnosed with any eye disease (such as macular degeneration, cataract, diabetic retinopathy, or glaucoma)? (2) Do you have amblyopia? (3) Did you receive an optometric or ophthalmic consultation within the last 6 months? (4) Did you experience a sudden vision loss or distortion? During the test, both eyes are tested consecutively, covering one eye at a time. The participant will wear his/her spectacles, if present. The participant is presented a sequence of optotypes (i.e., tumbling-E and triangle-circle optotypes) that the participant must correctly identify, in addition to various grate sizes, both near and at a distance. The Amsler grid test is performed to detect any macular problems.

#### Referral and intervention uptake

**Fig. 2:**
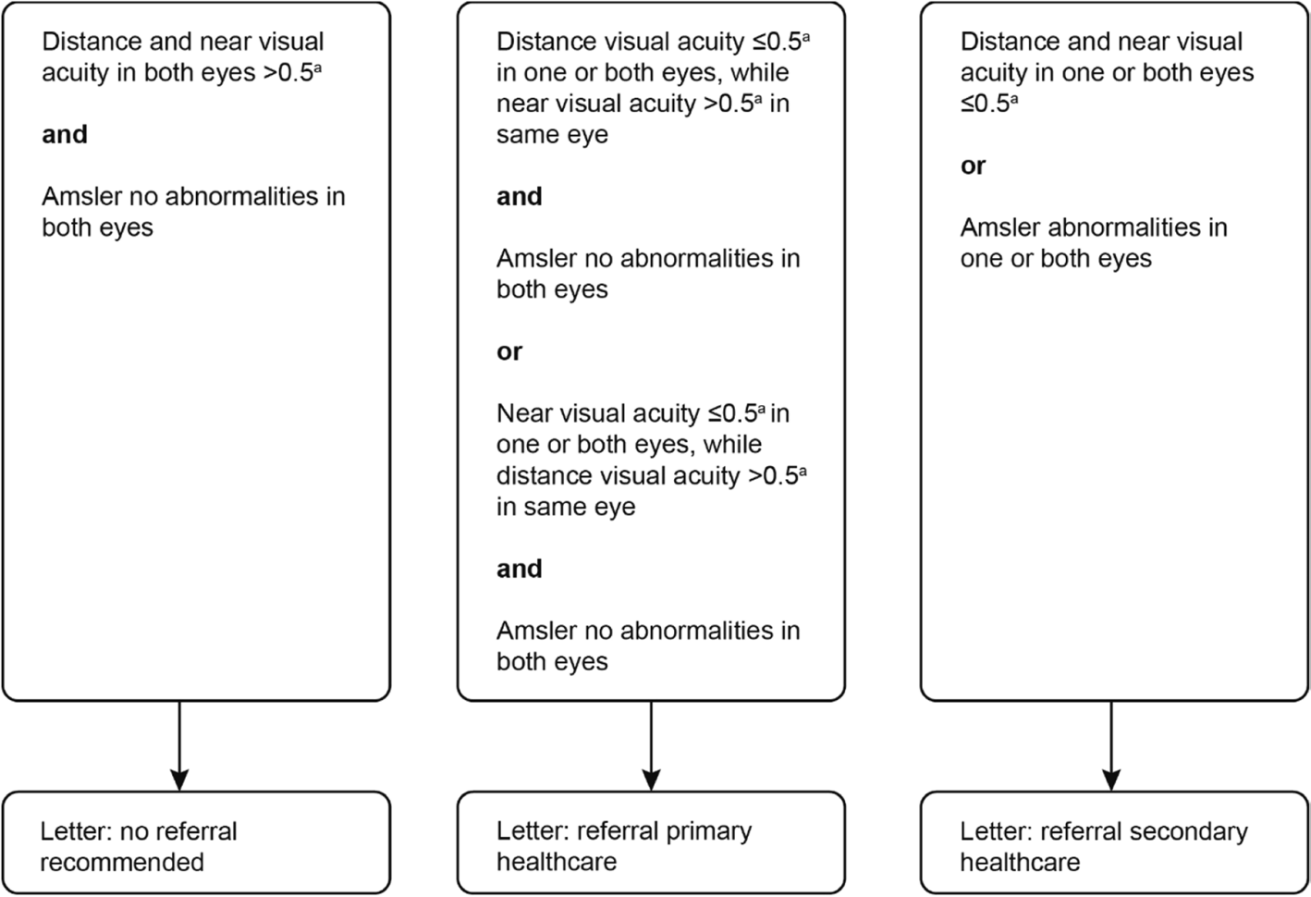
Flowchart referral ^a^Snellen visual acuity

The results of the eye screening will be checked by an optometrist and will be made available to the participant by letter within 1 week after the screening takes place. This will include a recommendation for a referral, if necessary. Participants will be referred to an optometrist (primary or secondary healthcare). In the Netherlands, primary healthcare provides the first point of contact in the healthcare system, accessible without referral. In the context of our study, primary healthcare encompasses optometrists who typically work at an optical store or at a community health center. Secondary eye care comprises clinical services which need a referral from a GP or primary care optometrist. The criteria for referral can be found in Fig. 2. These criteria were based on the classification of vision impairment of “The International Classification of Disease 11 (2018) of vision impairment” [36], which describes a vision impairment as having a visual acuity worse than 0.5 (20/40) (Snellen). In addition, the driving requirements of the government of the Netherlands [37] were used, where a visual acuity of at least 0.5 (20/40) (Snellen) is required for driving a car. The nurse will discuss the referral with the patient and will check whether the patient has used the referral. If not, additional motivational conversations between the involved community nurse and participant will take place after 2, 4, and 6 weeks. The optometrist who receives the referral will perform an optometric examination according to their usual care and practice guidelines, tailored by the information supplied in our referral. Throughout this RCT, the expenses for these examinations will be reimbursed.

#### Reliability online eye screening

Recently, we studied the reliability and feasibility of the online eye screening in patients receiving home healthcare [28]. Forty patients (80 eyes) were included. The following mean differences between the online eye screening and reference tests were found: distance visual acuity 0.02 logMAR, near visual acuity measured with tumbling-E optotypes 0.06 logMAR, and 0.03 logMAR with the triangle-circle optotypes. For distance visual acuity, 75% of the individual data points were within the non-inferiority threshold (± 0.15 logMAR). For near visual acuity, 51% and 58% were within the non-inferiority threshold for the tumbling-E and triangle-circle optotypes, respectively. The results showed negligible to small mean differences between the tests implemented in the online eye screening and the traditional visual acuity measurements. The agreement between tests for macular problems was 75% [28]. The convenience and ease of an online eye screening at home, along with the added support of a trusted community nurse, were cited as the main advantages by participants. However, the study also identified several disadvantages of an online eye screening at home, including the lack of optimal lighting conditions, and the limited scope of the screening. The online eye screening may not be able to detect certain eye conditions that can be identified through more comprehensive examinations performed by eye care professionals. Furthermore, several participants and community nurses commented on the duration of the eye screening, with a mean duration of 42 min. The online eye screening has been adapted, by removing the measurement of refractive error, to shorten the eye screening. To determine whether patients should be referred to an eye care professional, which is the goal of eye screening, visual acuity, and Amsler measurements should suffice.

Previously, the reliability and efficacy of the web-based tool were also tested against traditional subjective manifest refraction by an optometrist (golden standard) in a prospective open-label non-inferiority clinical trial in 200 eyes of 100 healthy volunteers (18–40 years), with a refraction error between -6 and + 4 diopters (non-inferiority criterion 0.5D or less) [38]. Web-based assessment was considered non-inferior to the reference test with an excellent intraclass correlation of 0.92. In addition, uncorrected visual acuity was also similar and significantly improved using the prescription obtained from the web-based tool.

Therefore, it was concluded that web-based eye testing is a valid and safe method for measuring visual acuity and refractive error in healthy eyes [38].

#### Training community nurses

Teams of community nurses, who have been randomized to the intervention group, will be trained to assist patients with the online eye screening. All nurses involved in the eye screening will administer the online eye screening twice with two voluntary participants (e.g., relatives, colleagues, or friends) before they start screening study participants. The training also teaches nurses how to motivate their patients for intervention uptake after referral, by making use of motivational interviewing. Motivational interviewing is an effective way to explore and resolve ambivalence that individuals may have about health behavior, with the goal of promoting positive change [39, 40].

### Care as usual

The intervention and control groups will receive CAU. The control group will not be actively screened with the Easee tool for eye complaints; all necessary care will be provided by the community nurse. It is possible that eye complaints are being discussed spontaneously. These are registered and/or followed up as usual by the nurse and home healthcare organizations.

### Study procedures

Measurements in both groups will take place at baseline (T0) and after 6 months (T1) and 12 months (T2) of follow-up (Table 2). A baseline assessment will take place before randomization. During all assessments, visual acuity measurements and Amsler tests will take place at the participants’ homes. Questionnaires will be conducted by telephone and immediately entered into Castor (data entry software) (Additional file 2).

**Table 2:**
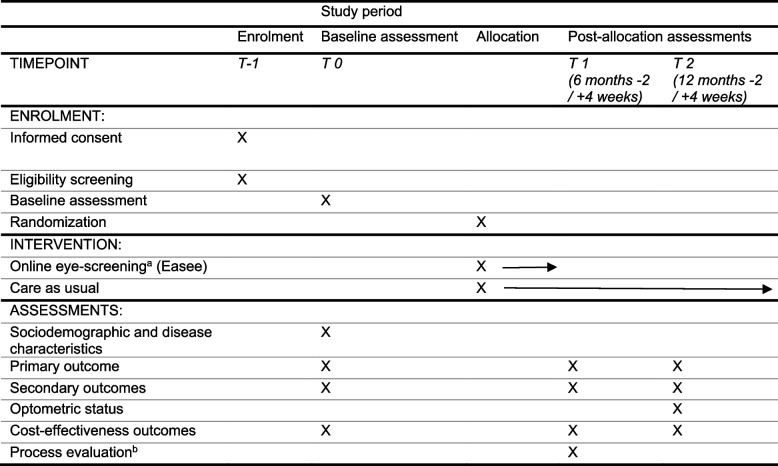
Study design and measurements

^a^After allocation, intervention group only

^b^Intervention group only

Visual acuity in the intervention group will additionally be measured with the online nurse-assisted eye screening after baseline/randomization.

Optometric status 12 months after baseline will include extensive optometric examination at the participants’ home with mobile equipment, including slit lamp examination for lens status, intraocular pressure (Icare), and fundus photography for thorough examination of the retina and optic nerve status (handheld Retcam). Any suspected deviation will be discussed with an ophthalmologist after which a patient will be referred. This measurement will be used for diagnostic purposes indicating whether we may have missed (latent) pathology in both groups but also to make sure that all participants will receive eye care after the study, if necessary.

### Outcome measures

Sociodemographic and disease characteristics, including reasons for receiving home healthcare, will be self-reported and based on medical records from the home healthcare organizations with the participant’s consent. Ophthalmic diagnoses and information on interventions will be obtained from the GP, optometrist, or ophthalmologist with the participant’s consent, for patients who received a referral.

#### Primary outcome

The primary outcome is a clinically relevant change of 2 lines or more (exceeding measurement error) on the Colenbrander-1 M visual acuity chart between baseline and 12 months follow-up.

Visual acuity will be assessed in both groups for both eyes separately at baseline and after 6 months and 12 months follow-up, by trained research assistants. These measurements will be performed in participants’ homes and will not be connected in any way to the nurse-assisted web-based screening, and outcomes will not be communicated with patients. Research assistants will be trained according to a strict protocol. The Colenbrander 1-M chart has an occluder attached to it with a rope of 1 m length. Participants will be asked to sit on a chair in front of the chart that is fixed on a music stand and adjusted to the participant’s height. The research assistant makes sure that there is sufficient light and no inappropriate reflection of light on the chart. Participants will be allowed to wear their own spectacles, except for reading addition. In glasses with varifocal lenses, participants will be asked not to look through the reading addition but straight over it. The occluder is equipped with a correction of + 1D. The number of letters read correctly will also be converted to logMAR visual acuity. The baseline and 6-month interpretations of the results of the vision-related measurements will not be shared with the participant and community nurse [41].

#### Secondary outcomes

The following vision-related outcomes will also be assessed: (1) average visual acuity change per eye in letters per participant between baseline and 12 months follow-up, including stenopeic visual acuity; (2) number of participants and eyes with baseline vision impairment (visual acuity 8/24 or lower with available optical correction) with clinically relevant progress of 2 lines or more; (3) optometric status 12 months after baseline; (4) vision-related quality of life with the EyeQ [42]; and (5) Amsler grid chart to detect symptoms of macular degeneration.

In addition, the following health-related measures will be assessed: (1) falling accidents and bone fractures with a shortened version of the “fall and fracture calendar” [7, 43]; (2) depressive symptomatology, measured with the Patient Health Questionnaire (PHQ-9) with nine questions corresponding to the Diagnostic Statistical Manual symptoms for major depressive disorder during the past 2 weeks. Good reliability and validity were shown using Rasch analysis [44, 45]; (3) health-related quality of life with the EuroQol 5-Dimension 5-Level questionnaire (EQ-5D-5L) which covers the dimensions mobility, self-care, daily activities, pain and discomfort, and anxiety and depression. Evidence indicated a reliable and valid instrument that describes health status [46, 47]; (4) well-being with the ICEpop CAPability measure for Older people (ICECAP-O) is a measure of capability in older people for use in economic evaluations. The ICECAP-O has good convergent validity with well-being measures [48]; (5) health literacy with the European Health Literacy Survey Questionnaire (HLS-EU-Q16). A high correlation (*r* = 0.82) was found with the general health literacy score of the HLS-EU-Q47 [49].

#### Cost-effectiveness

The Institute for Medical Technology Assessment (iMTA) Medical Cost Questionnaire (iMCQ) will be used to measure healthcare utilization at baseline and after 6 and 12 months of follow-up [50]. Standard costs for health care utilization from the Dutch costing manuals will be used [51]. In addition, the costs of the intervention will be measured, including treatment uptake after referral. Treatment uptake related to eye care will also be measured and valued for the control group. Medication use is valued using prices from Dutch Medical costs guidelines [52].

#### Process evaluation

For participants in the intervention group and community nurses, a process evaluation will take place at 6 months, by using a questionnaire. The RE-AIM framework (Reach, Effectiveness, Adoption, Implementation, and Maintenance) of the United Kingdom- Medical Research Council guidelines for process evaluations will be used. Three essential features of understanding the process through which outcomes are achieved will be identified: (1) context, (2) implementation, and (3) mechanisms of impact [31]. In addition, elements from the Telemonitoring Acceptance Framework [32] will be used to assess trust, behavioral intention, self-efficacy, performance expectancy, effort expectancy, social influence, facilitating conditions, technology anxiety, hedonic motivation, and user opinions regarding the nurse-assisted online eye-screening tool.

### Adverse events

All study-related adverse events (AEs) reported spontaneously by the subject or observed by the investigator or his staff will be recorded in Castor. Serious AEs (SAEs) will be reported through the web portal “ToetsingOnline” to the METC of Amsterdam UMC, which approves the protocol, within 7 days of first knowledge for SAEs that result in death or are life-threatening followed by a period of a maximum of 8 days to complete the initial preliminary report. All other SAEs will be reported within a period of a maximum of 15 days after the sponsor has first knowledge of the SAE. All SAEs will be followed until a stable situation has been reached or the SAE has resolved. Besides, the patient’s GP will be contacted.

### Data management

Data will be entered into Castor and converted into the statistical software packages SPSS, RStudio, and Stata. For each participant, a code (from 1000 to 3999) is used. A “key file” (in which these codes are linked with the patients’ names, addresses, and phone numbers) will be saved separately and will be deleted after the study has ended. Only the executive researchers will be able to access the key file. Data will be stored at Amsterdam UMC, location VUmc, computer network with a password in a folder that only the executive researchers can access. Medicine lists and signed application and consent forms will be kept in a locked cabinet in a locked room, only accessible to the executive researchers. Data will be stored for 15 years before they will be destroyed (Dutch law).

### Monitoring

Monitoring will be conducted by the clinical research bureau of Amsterdam UMC, location VUmc. The independent monitor will monitor the study data according to Good Clinical Practice. Informed consents will be checked in a selection of subjects. In addition, during the onsite monitoring, source data verification will be carried out. The intensity of this verification is related to the risk of the research, which is classified as negligible. The inclusion and exclusion criteria and the primary outcomes of the research will be checked. The monitor will also examine whether all (S)AEs have been adequately reported within the timelines as required by law and regulations.

### Statistical analysis

A table will be presented showing baseline demographic and clinical characteristics for each group.

To determine the validity of the questionnaires that are used within this study population, item response theory models will be performed. These models incorporate the characteristics of questionnaire items and responses, rendering a more accurate representation of the score on the latent construct, increasing the validity of the used questionnaires and the accuracy of the obtained results.

To determine effectiveness, linear mixed models (LMMs) will be used for continuous outcomes and logistic generalized estimating equation (GEE) analyses for dichotomous outcomes, given the longitudinal structure of the data. This will be based on intention-to-treat (i.e., all data will be included independent of completion of the intervention, including uptake after referral). We will use group (intervention vs. control), time, and the interaction between group and time as predictors. *P*-values less than 0.05 will be considered statistically significant.

The cost-effectiveness from a healthcare and societal perspective of eye screening compared to CAU will be determined based on the primary (incidence of clinically relevant changes in visual acuity) and secondary outcomes. Cost-utility will be determined with quality-adjusted life-years (QALYs) based on the EQ-5D-5L. Bias-corrected and accelerated bootstrapping with 5000 replications will be used to calculate 95% confidence intervals around the mean difference in total costs between the two groups for both perspectives. Incremental cost-effectiveness ratios (ICERs) and incremental cost-utility ratios (ICUR) will be calculated, as well as cost-effectiveness curves. Bootstrapping will be used to estimate the uncertainty surrounding the ICERs, which will be plotted graphically on cost-effectiveness planes. Cost-effectiveness acceptability curves will also be estimated. Costs for the consequences of not receiving ophthalmic treatment (for example, if the patient is not able to follow up on the referral because of other health problems) will be estimated and modeled. Findings will be integrated with published reports and literature to extrapolate the findings to a national level.

Guidelines by the ISPOR Task Force will be used for the budget impact analysis (BIA), i.e., relevant features of the health care system, access restrictions, anticipated uptake, and the use and effect of current and new intervention(s) will be considered. Also, the size and eligibility of the population, cost of interventions and treatment mixes (e.g., other ophthalmological or (mental) healthcare treatment), and changes expected in condition-related costs will be considered in the BIA. Sensitivity analyses will be performed using different scenarios from the perspective of decision-makers. Missing cost and effect data will be imputed using multiple imputation techniques according to the MICE algorithm.

## Discussion

To the best of our knowledge, this will be the first study to investigate the cost-effectiveness and cost-utility of a nurse-assisted online eye screening in home healthcare settings with the aim to reduce avoidable vision impairment in a fragile older population. It is of great importance that evidence of such an intervention is provided to lower the level of undiagnosed and untreated vision impairments in this population.

Timely diagnostics followed by timely treatment may improve patients’ visual functioning, health literacy, quality of life, and mental and physical health. It may increase independence and the ability to participate in social activities, thereby contributing to improve health [53]. Not only patients may benefit from online eye screening, but it may also lower healthcare costs. For instance, by reducing the higher risk of falls and fractures in visually impaired older adults [17] which may lead to a decreased need for care or nursing home admissions [1], which results in a lower economic burden [5].

We are aware that in the upcoming years, it is expected that the number of patients who will visit an ophthalmologist will increase, due to aging of the population. The National Institute for Public Health and the Environment has estimated that the absolute amount of visual disorders in the Netherlands will increase by 45% between 2018 and 2040 [4]. A good collaboration between caregivers is of the utmost importance to meet the demand of care. In our study, we will stimulate this by using a well-considered referral scheme and optimize the capabilities of optometrists to avoid an overload of patients for the ophthalmologists as a result of eye screening in home healthcare [54].

In addition, based on what Clarke et al. [11] mentioned about older adults often not following up on offered interventions after screening, motivational conversations between the involved nurse and participant will be part of the intervention to increase referral uptake. All nurses will be trained in motivational interviewing techniques, which have proven to be effective in previous settings, for example, in supporting medication adherence and stopping or preventing unhealthy behaviors [39, 40].

By making use of an online eye screening, instead of traditional visual acuity charts, participants do not have to leave their homes for an eye screening and will receive this screening from a nurse who they already know. This is especially relevant for this vulnerable population, which is often less mobile and may experience difficulties visiting care providers. Furthermore, online testing makes it possible to measure visual acuity at any point in time and due to the clear instructions, which provide guidance during the entire test, it is easy to use.

Besides the individual benefits, it should be mentioned that there are further advantages that justify the use of a digital test. The first and foremost reason is the scalability of a digital approach. A software approach is much easier to apply to any given number of patients. Second, test outcomes are recorded in a secured environment, which may be linked to the electronic record of the patient. This mitigates the risk of data losses and wrongful data entry. A digital test greatly expedites the collection of data for our current study, but also for future practice. Although online eye screening has advantages, a possible limitation will be the presence of a good Internet connection at the homes of this older population. However, this could potentially be solved by using a hotspot.

There may be some possible limitations to this study. The online eye screening will not incorporate any peripheral visual field testing. This way, we are not able to detect (early) cases of glaucoma or cerebrovascular causes of visual field loss. Performing any kind of visual field testing may increase the probability of finding an undiagnosed eye disease. However, the confrontation visual field examination can be considered gross and is difficult to perform [7]. Besides, most of the patients with abnormal visual function will be detected by only a near acuity test (84%), which was shown in a recent pilot study [7]. By only adding distance visual acuity, 91% of the referrals were captured [7]. However, to be certain we will not miss any eye pathology, participants in both groups will receive an optometric exam at 12 months.

The 12-month measurement is chosen for the purpose of measuring optometric status, to avoid influencing the behavior of participants and nurses regarding referral or intervention uptake in both groups during the course of the study, i.e., after baseline or 6 months. It can be argued whether it is ethical to wait 12 months to have that important information available (especially in participants in the control group, with a lower visual acuity measured). However, optometric screening as compared to online eye screening is much more expensive and probably not necessary for most patients. As an ethical decision, participants in both groups will receive the optometric examination at the 12-month measurement. We expect this to decrease the likelihood for selective non-response/drop-out in the control group as participants will receive eye care if needed.

We will use cluster randomization in which clusters of community nurses will be randomized rather than individual patients, to prevent contamination between those receiving the intervention and those who are not. However, if participants in the control group will actively look for eye care, due to contamination, this will come up in the iMCQ questionnaire [50]. A limitation of using cluster randomization lies in the fact that biased recruitment can occur. This can happen when a nurse recruiting a participant has both knowledge of the characteristics (vision) of the participant and the allocation schedule [55]. Therefore, randomization of the clusters will be done after the home healthcare organizations have invited participants.

It may be a challenge to find enough participants who want to participate in this study. Therefore, we work together with different home healthcare organizations and use multiple waves of inclusion. We will exclude patients with severe cognitive functioning. Vision testing in people with very low cognitive skills is known to be difficult; therefore, the screening will not be reliable in this population [56].

As emphasized above, the economic evaluation to ensure the relevance of eye screening has not yet been investigated. Given the increasing costs resulting from demographic aging and the significant economic burden associated with vision impairments, we believe performing a cost-effectiveness and cost-utility study in reducing eye problems is essential. If online eye screening is found to be cost-effective, a clear and tailored implementation plan should be developed.

## Trial status

Protocol version number 5, date 28-02-2023. Recruitment began on 23 March 2023 and will be completed in April 2024.

### Supplementary Information

**Additional file 1.** SPIRIT Checklist for Trials.

**Additional file 2.** World Health Organization trial registration dataset.

## Data Availability

The final dataset will be placed in an online repository upon completion of the study and will be available upon reasonable request.

## Abbreviations

WHO: World Health Organization
GP: General practitioner
RCT: Randomized controlled trial
CAU: Care as usual
UMC: University Medical Centers
SPIRIT: Standard Protocol Items: Recommendations for Interventional Trial
PHQ-9: Patient Health Questionnaire
EQ-5D-5L: EuroQol 5-Dimension 5-Level questionnaire
ICECAP-O: ICEpop CAPability measure for Older people
HLS-EU-Q16: European Health Literacy Survey Questionnaire
iMTA: Institute for Medical Technology Assessment
iMCQ: Medical Cost Questionnaire
RE-AIM: Reach, Effectiveness, Adoption, Implementation, and Maintenance
AEs: Adverse events
SAEsa: Serious adverse events
LMMs: Linear mixed models
GEE: Generalized estimating equation
QALYs: Quality-adjusted life-years
ICERs: Incremental cost-effectiveness ratios
ICUR: Incremental cost-utility ratios
BIA: Budget impact analysis

## References

[1] Keunen JEE, SnouckHurgronje M, OldeRikkert MGM, van Rens GHMB, Limburg JJ (2011). Een simpele oogtest voor slechtziende ouderen in zorginstellingen. A simple eye test for visually impaired elderly in healthcare institutions. Tijdschrift voor Ouderengeneeskunde..

[2] Burton MJ, Ramke J, Marques AP, Bourne RRA, Congdon N, Jones I (2021). the lancet global health commission on global eye health: vision beyond 2020. Lancet Glob Health.

[3] Bourne RRA, Flaxman SR, Braithwaite T, Cicinelli MV, Das A, Jonas JB (2017). Magnitude, temporal trends, and projections of the global prevalence of blindness and distance and near vision impairment: a systematic review and meta-analysis. Lancet Glob Health.

[4] Rijksinstituut voor Volksgezondheid en Milieu Ministerie van Volksgezondheid Welzijn en Sport. Trendscenario Ziekten en aandoeningen. https://www.volksgezondheidtoekomstverkenning.nl/c-vtv/trendscenario-update-2020/ziekten-aandoeningen. Accessed 11 Nov 2022.

[5] Alma MA, van der Mei SF, Melis-Dankers BJ, van Tilburg TG, Groothoff JW, Suurmeijer TP (2011). Participation of the elderly after vision loss. Disabil Rehabil.

[6] Ma N, Low S, Hasan S, Banna S, Patel S, Kalsi T (2023). Provision of eye care services and interventions in care homes: a narrative synthesis review. Eur Geriatr Med.

[7] van Nispen R, van der Aa H, Timmermans F, Meijer N, Koster N, de Blok J (2019). Reducing avoidable visual impairment in elderly home healthcare patients by basic ophthalmologic screening. Acta Ophthalmol.

[8] Limburg JJ, van Rens GHMB, Keunen JEE (2009). Visuele beperkingen bij ouderen in Nederland - risicogroepen en mogelijkheden tot interventie. Tijdschrift Gerontol Geriatr..

[9] Yamada Y, Vlachova M, Richter T, Finne-Soveri H, Gindin J, van der Roest H (2014). Prevalence and correlates of hearing and visual impairments in European nursing homes: results from the SHELTER study. J Am Med Dir Assoc.

[10] Tseng YC, Liu SH, Lou MF, Huang GS (2018). Quality of life in older adults with sensory impairments: a systematic review. Qual Life Res.

[11] Clarke EL, Evans JR, Smeeth L (2018). Community screening for visual impairment in older people. Cochrane Database Syst Rev..

[12] Evans BJ, Rowlands G (2004). Correctable visual impairment in older people: a major unmet need. Ophthalmic Physiol Opt.

[13] van der Aa HP, Comijs HC, Penninx BW, van Rens GH, van Nispen RM (2015). Major depressive and anxiety disorders in visually impaired older adults. Invest Ophthalmol Vis Sci.

[14] Gleeson M, Sherrington C, Keay L (2014). Exercise and physical training improve physical function in older adults with visual impairments but their effect on falls is unclear: a systematic review. J Physiother.

[15] de Boer MR, Pluijm SM, Lips P, Moll AC, Volker-Dieben HJ, Deeg DJ, van Rens GH (2004). Different aspects of visual impairment as risk factors for falls and fractures in older men and women. J Bone Miner Res.

[16] Liu S, Zhu Y, Chen W, Sun T, Cheng J, Zhang Y (2015). Risk factors for the second contralateral hip fracture in elderly patients: a systematic review and meta-analysis. Clin Rehabil.

[17] Feng YR, Meuleners LB, Fraser ML, Brameld KJ, Agramunt S (2018). The impact of first and second eye cataract surgeries on falls: a prospective cohort study. Clin Interv Aging.

[18] Harrison T, Guy S, Mackert M, Walker J, Pound P (2012). A study of the health literacy needs of people with visual impairments. Res Theory Nurs Pract.

[19] Flaxman SR, Bourne RRA, Resnikoff S, Ackland P, Braithwaite T, Cicinelli MV (2017). Global causes of blindness and distance vision impairment 1990–2020: a systematic review and meta-analysis. Lancet Glob Health.

[20] Blindness GBD, Vision Impairment C, Vision Loss Expert Group of the Global Burden of Disease S (2021). Causes of blindness and vision impairment in 2020 and trends over 30 years, and prevalence of avoidable blindness in relation to VISION 2020: the Right to Sight: an analysis for the Global Burden of Disease Study. Lancet Glob Health..

[21] Fricke TR, Tahhan N, Resnikoff S, Papas E, Burnett A, Ho SM (2018). Global prevalence of presbyopia and vision impairment from uncorrected presbyopia: systematic review, meta-analysis, and modelling. Ophthalmology.

[22] Limburg JJ, Smith ET, van der Horst FG, Gruntjes RAGJM, Verstraten PFJ, Bartels JAMJ, van Langen JMP (2014). Onnodigeslechtziendheid onder ouderen in zorginstellingen: lessen uit een interventieproject in de regio Den Bosch. Tijdschrift Gerontol Geriatr.

[23] Owsley C, McGwin G, Scilley K, Meek GC, Seker D, Dyer A (2007). Effect of refractive error correction on health-related quality of life and depression in older nursing home residents. Arch Ophthalmol.

[24] Owsley C, McGwin G, Scilley K, Meek GC, Seker D, Dyer A (2007). Impact of cataract surgery on health-related quality of life in nursing home residents. Br J Ophthalmol.

[25] Desapriya E, Subzwari S, Scime-Beltrano G, Samayawardhena LA, Pike I (2010). Vision improvement and reduction in falls after expedited cataract surgery systematic review and metaanalysis. J Cataract Refract Surg.

[26] The International Agency for the Prevention of Blindness (IAPB). Vision 2020. https://www.iapb.org/about/vision-2020/. Accessed 24 Nov 2022.

[27] CBS. Gezondheid en zorggebruik; persoonskenmerken, 2014–2021. updated 09–01–2023. https://www.cbs.nl/nl-nl/cijfers/detail/83005NED?q=thuiszorg#PersonenDieThuiszorgOntvingen_90. Accessed 11 Jan 2023.

[28] Elsman EBM, Lee SQ, van der Aa HPA, van Nassau F, Wisse RPL, Maarsingh OR (2023). The evaluation of an online nurse-assisted eye-screening tool in older adults receiving home healthcare. Ophthalmic Physiol Opt..

[29] Andermann A, Blancquaert I, Beauchamp S, Dery V (2008). Revisiting Wilson and Jungner in the genomic age: a review of screening criteria over the past 40 years. Bull World Health Organ.

[30] Chan AW, Tetzlaff JM, Gotzsche PC, Altman DG, Mann H, Berlin JA (2013). SPIRIT 2013 explanation and elaboration: guidance for protocols of clinical trials. BMJ.

[31] Glasgow RE, Vogt TM, Boles SM (1999). Evaluating the public health impact of health promotion interventions: the RE-AIM framework. Am J Public Health.

[32] Imhof T. Home telemonitoring acceptance for eye care patients. https://purl.utwente.nl/essays/823412020. Accessed 11 Jan 2023.

[33] Smeeth L, Fletcher AE, Hanciles S, Evans J, Wormald R (2003). Screening older people for impaired vision in primary care: cluster randomised trial. BMJ.

[34] van der Aa HPA, van Rens G, Bosmans JE, Comijs HC, van Nispen RMA (2017). Economic evaluation of stepped-care versus usual care for depression and anxiety in older adults with vision impairment: randomized controlled trial. BMC Psychiatry.

[35] Callahan CM, Unverzagt FW, Hui SL, Perkins AJ, Hendrie HC (2002). Six-item screener to identify cognitive impairment among potential subjects for clinical research. Med Care.

[36] World Health Organization. Blindness and vision impairment. https://www.who.int/news-room/fact-sheets/detail/blindness-and-visual-impairment. Accessed 10 Nov 2022.

[37] Overheid.nl. Regeling eisen geschiktheid. 2000. https://wetten.overheid.nl/BWBR0011362/2021-07-01. Accessed 10 Nov 2022.

[38] Wisse RPL, Muijzer MB, Cassano F, Godefrooij DA, Prevoo Y, Soeters N (2019). Validation of an independent web-based tool for measuring visual acuity and refractive error (the Manifest versus Online Refractive Evaluation Trial): prospective open-label noninferiority clinical trial. J Med Internet Res.

[39] Papus M, Dima AL, Viprey M, Schott AM, Schneider MP, Novais T (2022). Motivational interviewing to support medication adherence in adults with chronic conditions: systematic review of randomized controlled trials. Patient Educ Couns.

[40] Frost H, Campbell P, Maxwell M, O’Carroll RE, Dombrowski SU, Williams B (2018). Effectiveness of motivational interviewing on adult behaviour change in health and social care settings: a systematic review of reviews. PLoS One.

[41] Colenbrander A. Measuring vision and vision loss. Duane’s Clinical Ophthalmology; 2001. Chapter 51 (Volume 5).

[42] Rausch-Koster TP, Luijten MAJ, Verbraak FD, van Rens G, van Nispen RMA (2022). Calibration of the Dutch EyeQ to measure vision related quality of life in patients with exudative retinal diseases. Transl Vis Sci Technol.

[43] Pluijm SM, Smit JH, Tromp EA, Stel VS, Deeg DJ, Bouter LM, Lips P (2006). A risk profile for identifying community-dwelling elderly with a high risk of recurrent falling: results of a 3-year prospective study. Osteoporos Int.

[44] Kroenke K, Spitzer RL, Williams JB (2001). The PHQ-9: validity of a brief depression severity measure. J Gen Intern Med.

[45] Lamoureux EL, Tee HW, Pesudovs K, Pallant JF, Keeffe JE, Rees G (2009). Can clinicians use the PHQ-9 to assess depression in people with vision loss?. Optom Vis Sci.

[46] Versteegh MM, Vermeulen KM, Evers SMAA, de Wit GA, Prenger R, Stolk EA (2016). Dutch tariff for the five-level version of EQ-5D. Value Health.

[47] Feng YS, Kohlmann T, Janssen MF, Buchholz I (2021). Psychometric properties of the EQ-5D-5L: a systematic review of the literature. Qual Life Res.

[48] Makai P, Koopmanschap MA, Brouwer WB, Nieboer AA (2013). A validation of the ICECAP-O in a population of post-hospitalized older people in the Netherlands. Health Qual Life Outcomes.

[49] Storms H, Claes N, Aertgeerts B, Van den Broucke S (2017). Measuring health literacy among low literate people: an exploratory feasibility study with the HLS-EU questionnaire. BMC Public Health.

[50] Bouwmans C H-vRL, Koopmanschap M, Krol M, Severens H, Brouwer W. Handleiding iMTA Medical Consumption Questionnaire Rotterdam: Erasmus Universiteit; 2013.

[51] Costing tool or reference prices. https://www.imta.nl/tools/costing-tool/. Accessed 2 Mar 2023.

[52] Medicijnkosten.nl. https://www.medicijnkosten.nl/. Accessed 2 Mar 2023.

[53] Huber M, Knottnerus JA, Green L, van der Horst H, Jadad AR, Kromhout D (2011). How should we define health?. Brit Med J..

[54] Oogvereniging NOG, Optometristen Vereniging Nederland. Juiste oogzorg op de juiste plek. Passende zorg | Optometristen Vereniging Nederland (OVN) (optometrie.nl). Accessed 2 Mar 2023.

[55] Puffer S, Torgerson DJ, Watson J (2005). Cluster randomized controlled trials. J Eval Clin Pract.

[56] Campos JL, Hobler F, Bitton E, Labreche T, McGilton KS, Wittich W (2019). Screening for vision impairments in individuals with dementia living in long-term care: a scoping review. J Alzheimers Dis.

