# Supplementary material for: Health economic evaluation of a nurse-assisted online eye screening in home healthcare to reduce avoidable vision impairment (iScreen): study protocol for a cluster randomized controlled trial
Source: Trials. 2024 Feb 2;25:102. doi: 10.1186/s13063-023-07882-0 (PMC10835833; doi:10.1186/s13063-023-07882-0)
Supplement: Supplementary file 2 — Additional file 2. World Health Organization trial registration dataset. [file 13063_2023_7882_MOESM2_ESM.docx]

| **Data category** | **Information** |
| --- | --- |
| Primary registry and trial identifying number | ClinicalTrials.gov NCT06058637 |
| Date of registration in primary registry | 27 September 2023 |
| Secondary identifying numbers | NL78386.029.21 (protocol ID) |
| Source of monetary or material support | Dutch Organization for Health Research and Development program ‘Early Detection’ (ZonMw) |
| Primary sponsor | Amsterdam UMC, location VUmc |
| Contact for public/scientific queries | Vera Rooth: v.rooth@amsterdamumc.nl |
| Brief Title | iSCREEN-study: Online Nurse-assisted Eye-screening in Home Healthcare |
| Official Title | Online Nurse-assisted Eye-screening in Home Healthcare; Implementation Study and Economic Evaluation, from an Individual, Healthcare and Socio-political Perspective |
| Countries of recruitment | The Netherlands |
| Health condition(s) or problem(s) studied | Avoidable vision impairment |
| Intervention(s) | Eye screening on top of usual care |
| Key inclusion and exclusion criteria | Inclusion criteria   - Patients receive home healthcare for health problems - 65 years or older - Understanding of the Dutch language - Cognitive ability to participate in research (telephone assessment: six-item Mini Mental State Examination score >3)   Exclusion criteria   - Terminal illness, palliative home care - Cognitively unable to participate in research (e.g. late stage Alzheimer’s, Parkinson’s (telephone assessment: six-item Mini Mental State Examination score ≤3) - Having received an optometric or ophthalmic consultation within the last 6 months |
| Study type | - Interventional - Primary purpose: screening - Interventional study model: Parallel assignment - Masking: single (investigator) |
| Date of first enrolment | March 2023 |
| Target sample size | 240 |
| Recruitment status | Recruiting |
| Primary outcome(s) | Incidence in clinically relevant change of 2 lines or more on the Colenbrander-1M visual acuity chart |
| Key secondary outcomes | Vision related outcomes:   - Average visual acuity change per eye in letters - Number of participants with baseline vision impairment and clinically relevant change of 2 lines or more - Optometric status - Vision related quality of life with the EyeQ - Amsler grid   Health related outcomes:   - Fall and fracture calendar - PHQ-9 - EQ-5D-5L - ICECAP-O - HLS-EU-Q   Cost-effectiveness   - iMCQ |

**Table 3. World Health Organization Trial Registration Data Set**
